# A Novel Single-Step-Labeled ^212^Pb-CaCO_3_ Microparticle for Internal Alpha Therapy: Preparation, Stability, and Preclinical Data from Mice

**DOI:** 10.3390/ma14237130

**Published:** 2021-11-23

**Authors:** Ruth Gong Li, Kim Lindland, Tina Bjørnlund Bønsdorff, Sara Westrøm, Roy Hartvig Larsen

**Affiliations:** 1 Oncoinvent AS, 0484 Oslo, Norway; lindland@oncoinvent.com (K.L.); bonsdorff@oncoinvent.com (T.B.B.); westrom@oncoinvent.com (S.W.); sciencons@gmail.com (R.H.L.); 2 Institute of Clinical Medicine, University of Oslo, 0318 Oslo, Norway; 3 Department of Radiation Biology, Institute of Cancer Research, The Norwegian Radium Hospital, Oslo University Hospital, 0379 Oslo, Norway

**Keywords:** lead-212, alpha therapy, radionuclide therapy, radiopharmaceutical, calcium carbonate, microparticles, peritoneal carcinomatosis, micrometastasis

## Abstract

Lead-212 is recognized as a promising radionuclide for targeted alpha therapy for tumors. Many studies of ^212^Pb-labeling of various biomolecules through bifunctional chelators have been conducted. Another approach to exploiting the cytotoxic effect is coupling the radionuclide to a microparticle acting as a carrier vehicle, which could be used for treating disseminated cancers in body cavities. Calcium carbonate may represent a suitable material, as it is biocompatible, biodegradable, and easy to synthesize. In this work, we explored ^212^Pb-labeling of various CaCO_3_ microparticles and developed a protocol that can be straightforwardly implemented by clinicians. Vaterite microparticles stabilized by pamidronate were effective as ^212^Pb carriers; labeling yields of ≥98% were achieved, and ^212^Pb was strongly retained by the particles in an in vitro stability assessment. Moreover, the amounts of ^212^Pb reaching the kidneys, liver, spleen, and skeleton of mice following intraperitoneal (i.p.) administration were very low compared to i.p. injection of unbound ^212^Pb^2+^, indicating that CaCO_3_-bound ^212^Pb exhibited stability when administered intraperitoneally. Therapeutic efficacy was observed in a model of i.p. ovarian cancer for all the tested doses, ranging from 63 to 430 kBq per mouse. Lead-212-labeled CaCO_3_ microparticles represent a promising candidate for treating intracavitary cancers.

## 1. Introduction

**Figure 1:**
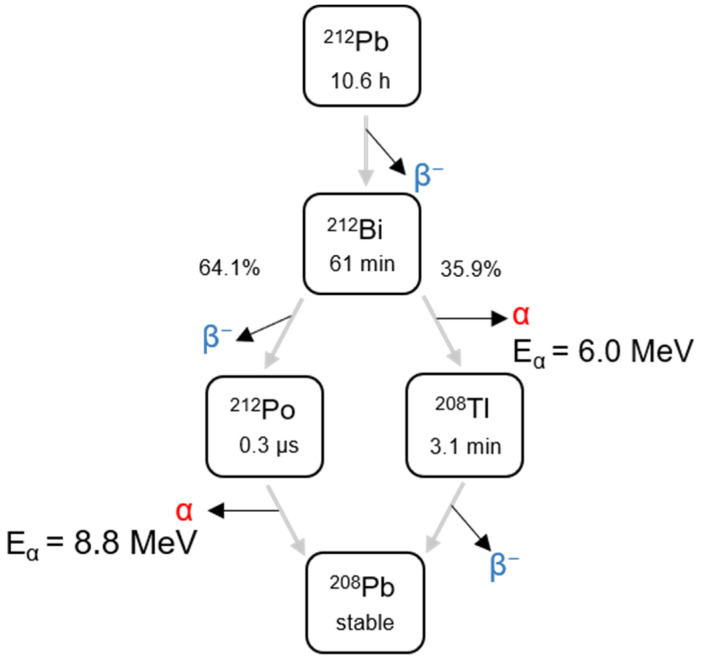
Decay scheme of ^212^Pb with associated alpha particle energy from its daughters.

The use of alpha-emitting radionuclides in cancer research and drug development is increasing [1]. This is due to their radiation characteristics, including high energy, short range, and high linear energy transfer with associated irreversible DNA damage, which make alpha particles superior to beta particles in the treatment of disseminated single cancer cells and micrometastases. Several alpha-emitters have been evaluated for therapeutic use in clinical trials: ^211^At, ^225^Ac, ^(223, 224)^Ra, ^227^Th, and ^213^Bi [1,2,3]. Lead-212, a beta emitter by itself, has attracted attention as an in vivo generator of alpha particles. Its first decay product, ^212^Bi, decays to either ^208^Tl (35.9%) via alpha emission, or to ^212^Po (64.1%), which undergoes subsequent alpha decay to stable ^208^Pb (Figure 1). Thus, one alpha particle is emitted per complete decay of ^212^Pb on average, which accounts for approximately 77% of the total energy released during decay.

The selection of the alpha-emitting radionuclide in the design of therapies depends on several factors. Among the important considerations are the physical half-life (*t*_1/2_) of the radionuclide, its chemical reactivity and stability with targeting moieties, the possibility of imaging it, and its availability. With this in mind, ^212^Pb represents a promising candidate. Its 10.6 h *t*_1/2_ is sufficiently long for the preparation of the radiopharmaceutical at or close to a hospital facility and shorter than most medically relevant alpha emitters (^225^Ac, ^(223, 224)^Ra, ^227^Th), which may be favorable in terms of treatment-related toxicity in some settings. For example, the infusion of carrier-bound ^212^Pb into body cavities (e.g., intraperitoneally) ensures that the radionuclide will mainly decay locally before reaching blood circulation due to its short *t*_1/2_. Further, ^212^Pb can be combined with targeting moieties by using several bifunctional chelators [4], can be imaged through its 238.6 keV gamma energy peak [5], and can be produced from longer-lived generators suitable for industrial-scale development [6,7].

Strategies for the specific delivery of the alpha emitter to its tumor target are considered paramount for ensuring efficient cancer cell kill and sparing normal tissues. Targeted alpha therapy (TAT) has been introduced for this purpose. Most often, TAT involves coupling the alpha-emitting nuclide to a targeting vector, such as a peptide or monoclonal antibody (mAb), through a bifunctional chelator. For ^212^Pb, the chelators TCMC and DOTA have been widely used for this purpose, and ^212^Pb[Pb]-DOTAMTATE and ^212^Pb[Pb]-TCMC-trastuzumab have been investigated in clinical trials for patients with SSTR+ metastatic neuroendocrine tumors and HER2-expressing tumors, respectively. Several other combinations with ^212^Pb have been reported in recent preclinical studies, including antibodies targeting HER1 [8,9,10], B7-H3 [11,12,13], CSPG4 [14,15], CD37 [16,17], CD38 [18], and PSMA-targeting ligands [19,20,21]. A challenge related to TAT using multi-step decaying radionuclides is the fate of the progenies in vivo. The radionuclide must be stably linked to the chelator and have a suitable pharmacokinetic profile. Moreover, the generation of alpha-emitting progeny during decay and associated recoil may cause dissociation of the daughter nuclide from the targeting complex. Although ^212^Pb does not experience alpha recoil, significant release of the daughter nuclide ^212^Bi from the chelator complex due to internal conversions has been measured experimentally [22,23]. In turn, the free daughter nuclide, which is often an alpha emitter itself, may be redistributed in vivo and cause adverse effects. 

An interesting approach to facilitating localized delivery of radionuclides is by passive targeting with microparticles or nanoparticles acting as carrier vehicles for the radionuclide or by combining a nanocarrier with a targeting agent [24]. In the absence of tumor-specific molecules, the carrier vehicle provides a means of retaining the radionuclide at the site of administration. Disseminated cancers in body cavities, such as peritoneal carcinomatosis and pleural effusions, may be suitable disease indications for such treatments. Furthermore, for ^212^Pb, a carrier is necessary to reduce the toxicity caused by unbound radionuclides. Systemically available lead and daughter bismuth are both known to be excreted via the kidneys [25,26], and lead may also cause bone marrow toxicity [27]. Previous work on ^212^Pb-labeled nanoparticles includes liposomes [28,29] and fullerene C60 nanoparticles [30]. However, limited in vivo data have been published [30,31]. For microparticles, data on ^212^Pb-labeled sulfur colloids and ferrous hydroxide microparticles in animal models of disseminated ovarian cancer were published in 1989 [32,33]. More recently, our group proposed calcium carbonate microparticles as carriers of the parent nuclide ^224^Ra, which are advantageous as a non-toxic, biodegradable, and biocompatible material [34,35,36,37,38]. These ^224^Ra-labeled calcium carbonate microparticles (Radspherin^®^) are currently in phase I clinical trials for patients with metastatic ovarian or colorectal cancer [39,40]. As our previous work has shown that significant amounts of ^212^Pb are retained on the CaCO_3_ particles following the decay of ^224^Ra [34,36,38], it was of interest to evaluate the feasibility and qualities of CaCO_3_ microparticles exclusively labeled with ^212^Pb. Compared to ^224^Ra-CaCO_3_ microparticles, ^212^Pb[Pb]-CaCO_3_ (abbreviated as ^212^Pb-CaCO_3_) microparticles could enable a faster production process and different pharmacokinetic and pharmacodynamic properties due to the considerably shorter *t*_1/2_ of ^212^Pb (10.6 h compared to the 3.6 days of ^224^Ra). Alpha radiation can be delivered in a shorter time frame and at a higher dose rate for most of the alpha dose when using ^212^Pb compared to ^224^Ra. 

In this work, we evaluated various CaCO_3_ microparticles for radiolabeling with ^212^Pb, including their physicochemical properties, radiolabeling yields, and in vitro stability. We have also investigated the biodistribution and therapeutic efficacy of ^212^Pb-labeled CaCO_3_ microparticles after intraperitoneal (i.p.) administration to mice with or without tumors.

## 2. Materials and Methods

### 2.1. Preparation of CaCO_3_ Particles

Calcium carbonate particles were produced following two different procedures to create particles with two distinct size populations. The CaCO_3_ microparticles of the first type (CaCO_3_ MPs) were produced following a procedure detailed elsewhere [38], which was identical to that for the CaCO_3_ particles used for ^224^Ra-labeling in Radspherin^®^—that is, following good manufacturing practices. The second type of CaCO_3_ particles were similar to the first, but glycerol was added during the spontaneous precipitation reaction to produce smaller microparticles (CaCO_3_ SMPs) as a result of increased solution viscosity [41,42]. Before mixing 1 M solutions of CaCl_2_ (Merck Group, Darmstadt, Germany) and Na_2_CO_3_ (Merck Group, Darmstadt, Germany), glycerol (Sigma-Aldrich, St. Louis, MO, USA) was added to each solution at a concentration of 50% (*v*/*v*), diluting the solutions to 0.5 M. The two solutions were then combined under vigorous stirring with an overhead stirrer (Eurostar 20; IKA^®^-Werke GmbH & Co. KG, Staufen, Germany) operating at 6000 RPM for 30 min. The resulting precipitate of CaCO_3_ SMPs was washed three times with water for injection (WFI) and then dried at 180 °C.

Sterile suspensions of CaCO_3_ MPs and CaCO_3_ SMPs were prepared by first washing the dry particles with WFI and ultrasonicating for improved dispersion, followed by suspension in 0.9% NaCl (Fresenius Kabi AG, Bad Homburg, Germany), and autoclaving at 121 °C for 20 min in a sealed crimp neck headspace vial. A size-controlling additive was necessary to inhibit the recrystallization and growth of CaCO_3_ [38], and the phosphonate pamidronate was evaluated for this purpose. Dissolved pamidronate disodium heptahydrate (MedKoo Biosciences, Inc., Morrisville, NC, USA) in 0.9% NaCl was added to the suspension before autoclaving at a concentration of 1% (*w*/*w*) with respect to CaCO_3_. In contrast to our previous work with ethylene diamine tetra(methylenephosphonic acid) (EDTMP), pamidronate was explored as an alternative due to the known complexation of EDTMP with ^212^Pb [38,43,44]. Pamidronate was substituted by EDTMP (Tokyo Chemical Industry Co., Ltd., Tokyo, Japan) in one experiment that involved radiolabeling with ^212^Pb to compare the labeling yields. The potential complexation between pamidronate and ^212^Pb was evaluated for varying pamidronate concentrations (0.01–8 mg/mL) using instant thin layer chromatography (ITLC) strips, a system in which chelated ^212^Pb migrates with the mobile phase, while >90% of unbound ^212^Pb remains at the origin line [38].

### 2.2. Characterization of CaCO_3_ Particles

The physicochemical characterization of the various CaCO_3_ particles was mainly performed by the National Physical Laboratory (Teddington, UK). The SMPs and MPs were analyzed either as dry raw material or after drying autoclaved suspensions with or without the phosphonate additive. Particle morphology and size were evaluated by scanning electron microscopy (SEM; Zeiss Auriga Focused Ion Beam SEM; Zeiss AG, Oberkochen, Germany) using a 3-kV accelerating voltage. Chemical composition analysis was performed by energy dispersive X-ray spectroscopy (EDX) with an EDX detector (Oxford Instruments X-Max; Oxford Instruments, Abingdon, UK) coupled to the SEM and using a 10-kV accelerating voltage. Crystalline composition was evaluated using X-ray diffraction analysis (XRD; Siemens D5000, Siemens AG, Munich, Germany) and by comparing the resulting diffraction patterns to vaterite, calcite, and aragonite reference spectra from the International Centre for Diffraction Data database. The relative composition of each polymorph was estimated based on the peak heights.

A second method for measuring the sizes of MPs and SMPs, including both the raw material and particles in a suspension with or without pamidronate, was employed using laser diffraction (Mastersizer 3000; Malvern Instruments Ltd., Worcestershire, UK) after dispersing the particles in purified water [38]. This method was used as quality control of various CaCO_3_ batches and to evaluate the over-time stability of particles in suspension.

### 2.3. Radiolabeling CaCO_3_ Particles with ^212^Pb

Lead-212 was obtained from a novel ^224^Ra/^212^Pb generator based on ^220^Rn emanation [45]. In brief, the generator exploited the emanation of the gaseous ^220^Rn daughter from a fixed ^224^Ra source in a closed vial, with subsequent decay to ^212^Pb and adsorption to the interior surface of the container. The ^224^Ra source-holding material was placed in such a way as to prevent any contact with the interior surface, which would have led to cross contamination. After separating the ^224^Ra source holder from the vial, ^212^Pb was isolated from the surface by washing with 0.1 M HCl (Honeywell, Charlotte, NC, USA), and the obtained solution was adjusted to a pH of approximately 7 with NaOH (VWR International, Radnor, PA, USA), NH_4_OAc (Merck Group, Darmstadt, Germany), or NaOAc (Merck Group, Darmstadt, Germany).

**Figure 2:**
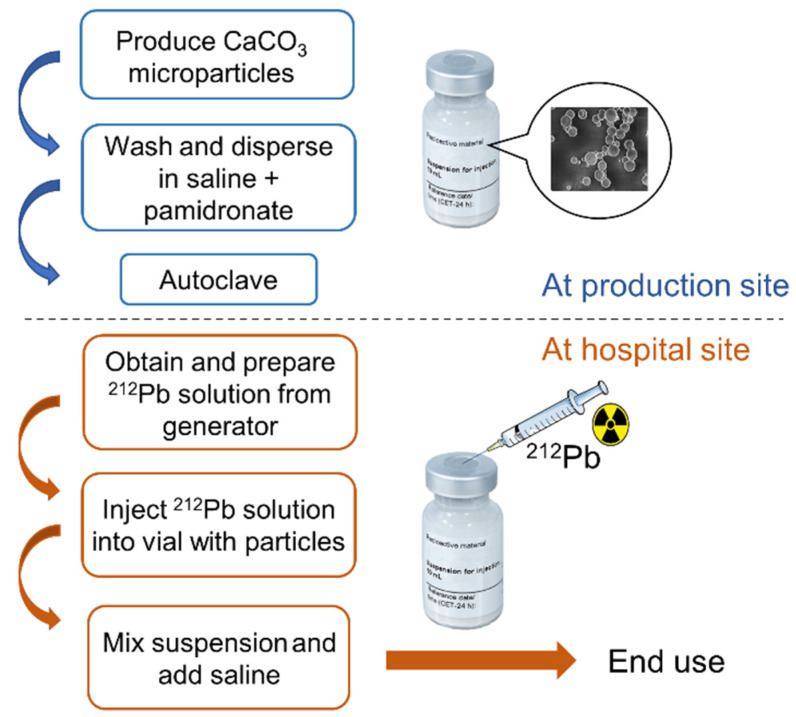
Scheme of the production process of ^212^Pb-labeled CaCO_3_ microparticles, showing the steps that can be performed on-site by the end user.

A single-step-labeling protocol was established to develop a fast procedure that can be performed at a nuclear medicine hospital facility. The labeling procedure involved injecting a sterile filtered ^212^Pb solution directly into the sealed headspace vial of autoclaved CaCO_3_ MP or CaCO_3_ SMP suspension with a syringe, resulting in adsorption of the ^212^Pb on the particle surface. The suspension was vortexed and placed on an orbital shaker operating at 225 RPM for 3–60 min. Finally, 0.9% NaCl was injected into the vial to achieve the desired isotonicity, radioactivity, and particle concentration. A flow chart of the production process is shown in Figure 2.

### 2.4. Evaluation of Radiolabeling 

Quality control measurements of the radiolabeled CaCO_3_ microparticles included the determination of radioactivity concentration, radiochemical yield/purity, over-time-stability, and possible breakthrough of the parent nuclide ^224^Ra. The radioactivity levels of the ^212^Pb solution and the vial of labeled CaCO_3_ particles in suspension were measured using an ionization chamber dose calibrator (Mirion Technologies (Capintec), Inc., Florham Park, NJ, USA) with a dial setting suitable for ^212^Pb [45]. The radiochemical yield (RCY) was determined by separating a small aliquot of suspension into particle fraction P and supernatant fraction S by centrifugation to measure the portion of ^212^Pb adsorbed on the particles. The two fractions were measured separately on an automatic gamma counter (Hidex Automatic Gamma Counter; Hidex Oy, Turku, Finland), and ^212^Pb was quantified by counts in the 60–110 keV window [45]. Finally, the RCY was calculated as the ratio of CPM(P) to CPM (P + S), where CPM denotes counts per minute in each fraction. In this setting, the RCY and the radiochemical purity (RCP) were identical quantities. In the following sections, the term “RCP” will be used to denote the generalized fraction of adsorbed ^212^Pb on the particles at a given time-point. The potential breakthrough of the ^224^Ra parent was assessed by measuring an aliquot of the ^212^Pb solution used for radiolabeling on a high-purity germanium detector (Mirion Technologies (Canberra), Inc., Atlanta, GA, USA), which could quantify the radionuclide directly from its 241-keV signal. 

In vitro stability was defined as the percentage of ^212^Pb retained on the microparticles after autoclaved suspensions were diluted 4 or 12.5 times in an isotonic infusion solution (Plasmalyte; Baxter International, Inc., Deerfield, IL, USA) with a pH of approximately 7 and supplemented with human serum albumin (10 g/L; Sigma-Aldrich, St. Louis, MO, USA). Dilution was performed from an initial CaCO_3_ concentration of approximately 25 mg/mL down to 2 or 6 mg/mL, and the samples were incubated in an oven set to 37 °C for 1.5–21 h. The diluted particles were separated from the incubation solution, and radioactivity in the two parts was measured as described above for the determination of the RCY and RCP.

### 2.5. Animals 

All procedures involving animals complied with national and EU regulations. The procedures were approved by the Institutional Committee on Research Animal Care (Department of Comparative Medicine, Oslo University Hospital, Oslo, Norway) and the Norwegian Food Safety Authority. In vivo experiments were performed in nude athymic mice (Hsd: Athymic Nude-*Foxn1^nu^*, Department of Comparative Medicine, The Norwegian Radium Hospital, Oslo University Hospital, Oslo, Norway) 5–9 weeks of age. 

### 2.6. Biodistribution 

The biodistribution of ^212^Pb-CaCO_3_ MPs was evaluated after i.p. administration to tumor-free mice and compared to i.p. administration of unbound ^212^Pb^2+^ in 0.9% NaCl (^212^PbCl_2_). A dose of 5 mg of ^212^Pb-CaCO_3_ MPs with a volume-based median particle diameter of 5 µm was investigated. The mice were sacrificed by cervical dislocation 2, 6, and 24 h after the treatment, and tissue samples were collected for radioactivity measurements and the calculation of the percent injected dose per gram (% ID/g) of tissue. A second experiment was conducted to compare the biodistribution of 5 mg of ^212^Pb-CaCO_3_ MPs and SMPs. To accurately determine the injected dose, three standard samples corresponding to 50% of the injected dose were used, and all radioactivity measurements were performed using Hidex automatic gamma counter with an appropriate calibration factor to obtain becquerel data [45]. The measurements were decay-corrected using the 10.6 h *t*_1/2_ of ^212^Pb. Kidney and liver samples were remeasured after one day of decay to ensure transient equilibrium between ^212^Pb and daughters. This was done to account for a possible contribution from daughter nuclides in the 60–110 keV window. For example, ^212^Bi is known to accumulate in these tissues in addition to ^212^Pb. 

A *t*-test was performed on each pair of experimental groups for each tissue to determine statistical significance, with the *p*-values adjusted according to the Holm–Sidak method for multiple comparisons and *p* < 0.05 considered the significance threshold. 

### 2.7. Therapeutic Efficacy

A pilot study of the therapeutic efficacy of ^212^Pb-CaCO_3_ MPs in the treatment of cavitary cancers was performed in an i.p. xenograft model of ovarian cancer in three separate experiments. Nude mice were inoculated intraperitoneally with 300,000 ES-2 cells (ATCC, Wesel, Germany) and treated with a single i.p. dose of 2–5 mg and 63–430 kBq of ^212^Pb-CaCO_3_ MPs the day after. The importance of the CaCO_3_ MP as a carrier vehicle was investigated by treating two of the groups with 129 or 268 kBq of ^212^PbCl_2_. All radioactivity doses were measured and calculated retrospectively from standard samples as described above. In each experiment, a control group of animals was given saline. In one experiment, a group was given 5 mg of non-labeled CaCO_3_ MPs suspended in saline and 1% (*w*/*w*) pamidronate. The mice were sacrificed by cervical dislocation upon reaching pre-determined disease-related end points, including severe body weight loss and/or buildup of ascites. The study was concluded, and remaining animals were censored at the timepoint corresponding to three times the median survival time of the longest surviving group. Statistically significant differences between the resulting survival curves were evaluated using GraphPad Prism software (GraphPad Software, San Diego, CA, USA). The Gehan–Breslow–Wilcoxon method was employed, and the obtained *p*-values were adjusted using the Holm–Sidak method for multiple comparisons with a significance threshold of *p* < 0.05.

Acute treatment-related toxicity was assessed by monitoring the body weight and general condition of the mice at regular intervals from the start of the study. 

## 3. Results

### 3.1. Stabilization of CaCO_3_ MPs and SMPs by Pamidronate

Calcium carbonate is metastable in an aqueous solution due to continuous dissolution and re-precipitation. Therefore, it was necessary to use an additive to prevent the recrystallization and growth of CaCO_3_ microparticles after autoclaving the particles in a suspension [38]. Pamidronate was studied for this purpose. To determine its suitability as an additive for ^212^Pb-labeled particles, possible complexation of ^212^Pb by pamidronate was ruled out for pamidronate concentrations ranging from 0.01 to 8 mg/mL on ITLC strips. Less than 1% of the ^212^Pb migrated with the mobile phase after correcting for unspecific ^212^Pb^2+^ migration in 0.9% NaCl. 

**Figure 3:**
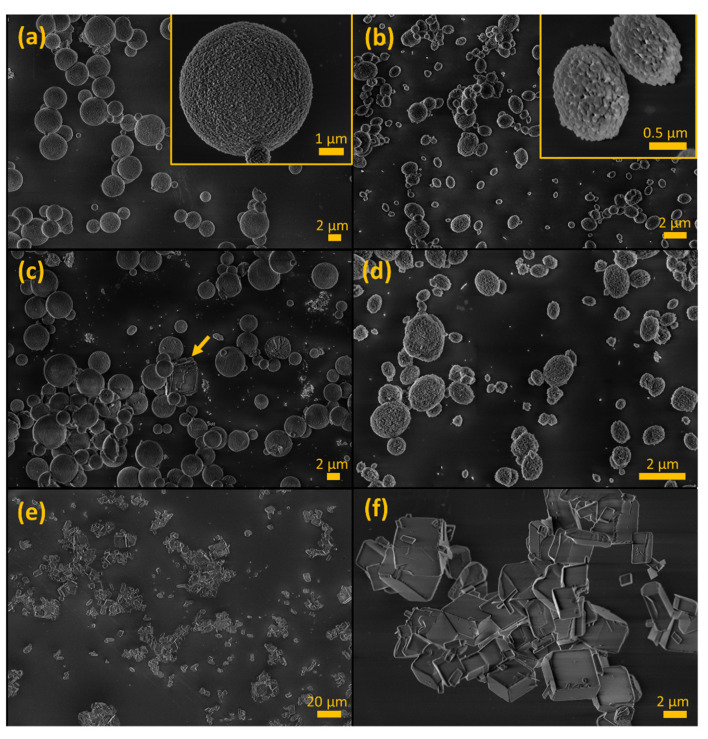
Scanning electron micrographs of various CaCO_3_ particles. (**a**) First type of CaCO_3_ microparticles (MPs), (**b**) smaller CaCO_3_ microparticles (SMPs) produced in glycerol presence, (**c**) autoclaved CaCO_3_ MPs with pamidronate (the arrow indicates a cuboidal particle), (**d**) autoclaved CaCO_3_ SMPs with pamidronate, and (**e**,**f**) autoclaved CaCO_3_ MPs with no additive.

**Figure 4:**
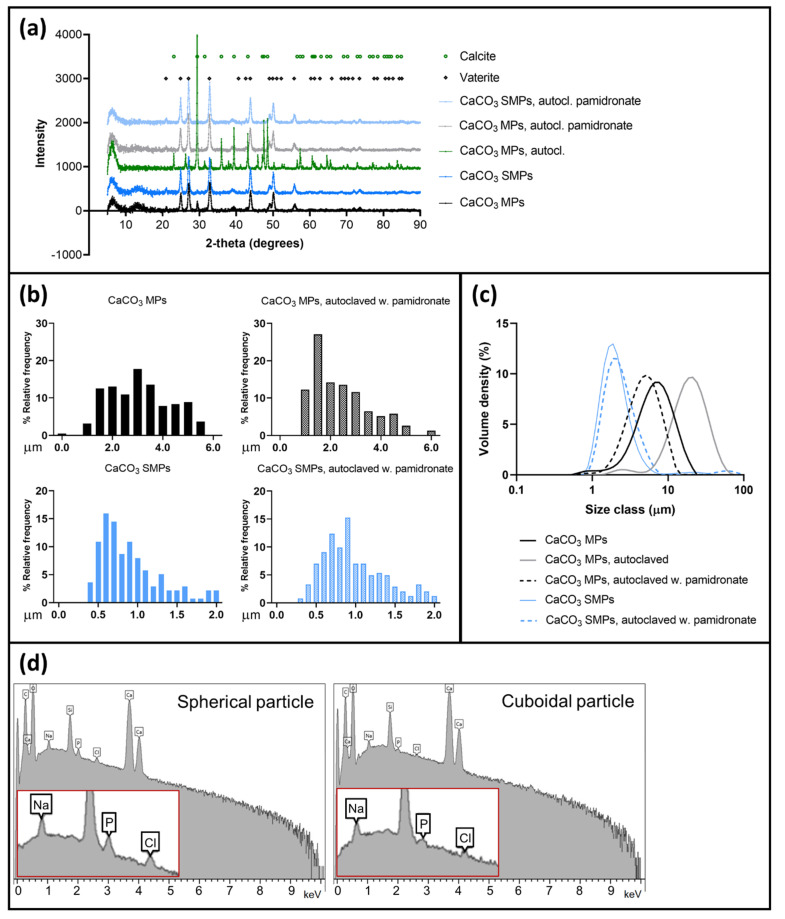
Physicochemical properties of various CaCO_3_ particles. (**a**) X-ray diffraction spectra of various CaCO_3_ particles. (**b**) Particle size distributions based on the circular or circle-equivalent diameters obtained from SEM images. (**c**) Particle size distributions based on laser diffraction. (**d**) Examples of EDX spectra of spherical and cuboidal particles from a sample of autoclaved CaCO_3_ MPs with pamidronate. The Si peak originated from the silicon substrate on which the sample was placed.

The stabilizing power of pamidronate was demonstrated using SEM imaging, XRD analysis, and laser diffraction when comparing samples of autoclaved particles in suspensions prepared with or without the additive, to their respective raw materials. Raw material samples of CaCO_3_ MPs were porous and spherical (Figure 3a), whereas CaCO_3_ SMPs were porous and prolate spheroid–shaped (Figure 3b). Both indicated the metastable vaterite form. The morphology of these particles remained unchanged after autoclaving them in a suspension with 1% (*w*/*w*) pamidronate (Figure 3c,d). Conversely, in the absence of pamidronate, large clusters of polygonal particles with cubic facets were observed, indicating transformation to the stable calcite form (Figure 3e,f). X-ray diffraction spectra of the various particles confirmed the presumed polymorphs (Figure 4a). The spherical or ellipsoidal MPs and SMPs that included pamidronate-stabilized particles consisted of at least 96% vaterite, with the remaining fraction being identified as calcite. The autoclaved MPs without pamidronate recrystallized to a composition of 87% calcite and 13% (several small peaks in the spectrum) originating from an unidentifiable phase that did not coincide with any of the CaCO_3_ polymorphs including aragonite. In general, a few polygonal particles were also observed in the vaterite samples of SMPs and MPs (example indicated in Figure 3c), in agreement with the small fraction of calcite detected by XRD.

The sizes of the various CaCO_3_ particles were measured either manually, using at least 100 individual particles in the SEM images (Figure 4b), or by laser diffraction (Figure 4c), which is a volume-based method that does not distinguish individual particles from dimers or larger clusters. As expected, laser diffraction showed larger particle sizes than SEM, especially for the recrystallized particles, where particle clustering was most severe. With both methods, the size of CaCO_3_ SMPs was consistently smaller than that of MPs. The modal diameter of SMPs was approximately 0.8 μm (volume-based median diameter of 2 μm), whereas that of MPs was 3 μm (volume-based median diameter of 5 μm). This confirmed the possibility of using glycerol in the production process to obtain smaller particles [41,42]. The size of recrystallized particles could not be satisfactorily evaluated using SEM because of the high degree of clustering. However, an average volume-based median diameter of 23 μm (*n* = 4 batches) was measured by laser diffraction, which is similar to previously reported data [38]. The sizes of both MPs and SMPs autoclaved with 1% (*w*/*w*) pamidronate corresponded to their respective raw materials and remained stable for particles in suspensions for up to two weeks.

One sample of autoclaved dried suspension of CaCO_3_ MPs with 1% (*w*/*w*) pamidronate was analyzed using EDX (Figure 4d), where the main signals originated from C, O, and Ca. Additionally, weak signals were detected from Na, Cl, and P, attributed to dried NaCl and pamidronate, which were the only sources of these elements in the sample, respectively. The detection of P indicates that pamidronate was present on the dried CaCO_3_ MP. The P peak height decreased for cuboidal particles compared to spherical particles, while Ca, C, and O remained similar. This suggested a higher concentration of pamidronate adsorbed to vaterite particles than to recrystallized calcite particles, confirming its stabilizing properties.

### 3.2. Single-Step-Labeling of ^212^Pb-CaCO_3_ Particles: Yield and Stability

**Figure 5:**
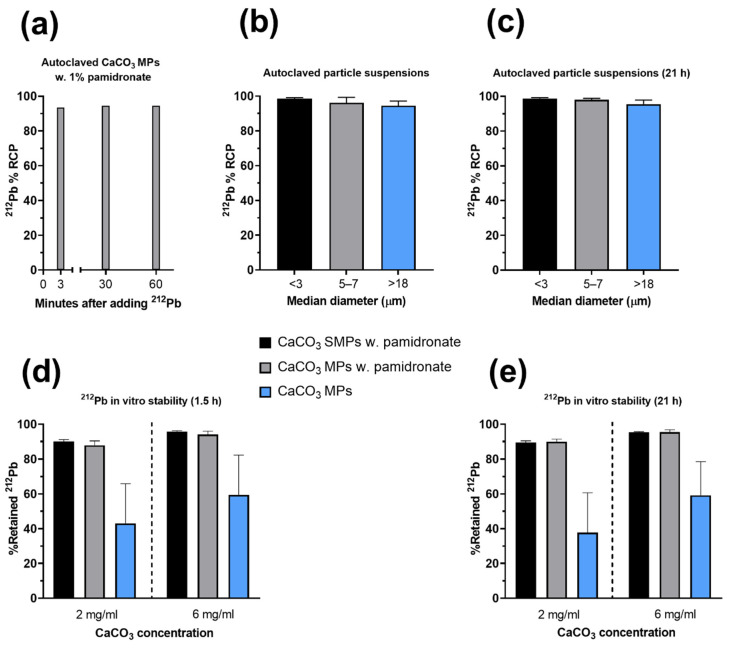
Radiochemical properties of various CaCO_3_ particles in suspension after autoclaving and labeling with ^212^Pb. The median diameters are based on laser diffraction measurements. (**a**) Percentage radiolabeling yield (same quantity as radiochemical purity (RCP)) as function of time after ^212^Pb was added (*n* = 2). (**b**,**c**) Percentage RCP as function of particle size or polymorphism (pamidronate-stabilized vaterite or recrystallized calcite) (**b**) on the same day as labeling and (**c**) after at least 21 h (*n* = 4–6). (**d**,**e**) Retained ^212^Pb on the particle types in (b, c) after 4× or 12.5× dilution and incubation in an isotonic solution with human serum albumin (*n* = 4).

The labeling procedure of CaCO_3_ particles involved a single injection of a sterile ^212^Pb solution into the sealed vial of particles in suspension with no co-reactants or subsequent processing of the suspension, apart from mixing the contents and final dilution with 0.9% NaCl to the desired concentration. Gamma spectroscopy measurements using a high-purity germanium detector did not detect any breakthrough of ^224^Ra in the ^212^Pb solution. The yield of the procedure—that is, the fraction of adsorbed ^212^Pb (RCP)—was evaluated after 3, 60, and 90 min of orbital rotation of the suspension after the radioactive solution was added. It was found that 94% of the ^212^Pb was adsorbed on CaCO_3_ MPs after as little as 3 min, with a negligible difference when the duration of agitation was extended (Figure 5a). In other experiments, a labeling yield of 98% on average was achieved for MPs and SMPs after 3 min of orbital rotation (*n* = 11; Figure 5b), whereas the labeling yield of recrystallized calcite particles was 95% on average (*n* = 4; Figure 5b). The RCP remained stable over two physical half-lives of the radionuclide (Figure 5c). In an experiment in which EDTMP was used in place of pamidronate, the labeling yield was considerably reduced to 66%.

The stability of various ^212^Pb-labeled CaCO_3_ particles was evaluated in vitro. The evaluation involved the incubation of the particles in an isotonic solution with human serum albumin at 4× or 12.5× dilution (6 mg/mL and 2 mg/mL, respectively) at approximately 37 °C. While all the variants had a ^212^Pb RCP of at least 90% in these experiments (Figure 5b,c), the incubation resulted in the release of ^212^Pb into the solution, which appeared to depend on the polymorphism and particle concentration (Figure 5c). The stability of the vaterite MPs and SMPs with pamidronate was the highest, with at least 88% ^212^Pb retained, whereas the recrystallized calcite particles released more than half of the initially adsorbed ^212^Pb after incubation at 2 mg/mL (Figure 5d,e). The stability of all particle types appeared to increase when they were incubated at 6 mg/mL than at 2 mg/mL, likely because of increased dissolution of CaCO_3_ at lower concentrations. Prolonging the duration of the incubation from 1.5 to 21 h did not affect the stability of any of the particles (Figure 5e).

### 3.3. Biodistribution of ^212^Pb

**Figure 6:**
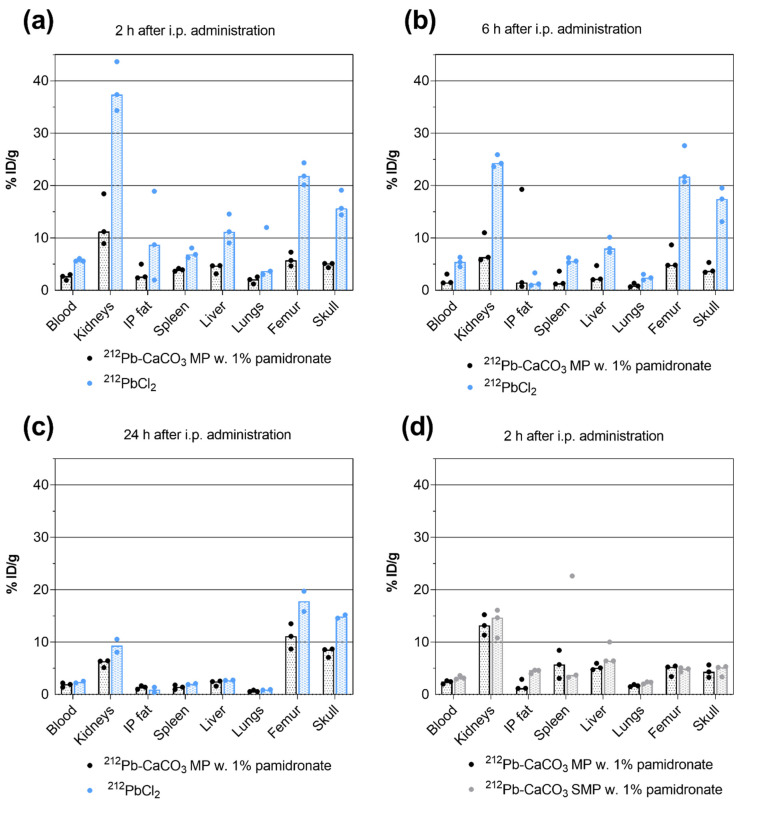
Biodistribution of ^212^Pb evaluated as the percentage of injected dose per gram (% ID/g) of tissue at a designated time following intraperitoneal (i.p.) administration. The bars represent the medians, and the symbols represent individual animals. (**a**–**c**) ^212^Pb-CaCO_3_ MPs compared with unbound ^212^Pb^2+^ administered as ^212^PbCl_2_, in terms of % ID/g of tissue after (**a**) 2 h, (**b**) 6 h, and (**c**) 24 h. (**d**) Data from the study comparing the biodistribution of ^212^Pb-CaCO_3_ MPs to that of SMPs produced in the presence of glycerol.

The biodistribution of 5 mg of ^212^Pb-CaCO_3_ MP was compared to that of unbound ^212^Pb^2+^ (^212^PbCl_2_) after i.p. administration of a 40–44-kBq dose of either compound. High levels of ^212^Pb could be detected in the kidneys, femur, and skull 2 h after ^212^PbCl_2_ administration. The % ID/g was significantly reduced in those tissues and in the blood, liver, and spleen for the MP-bound ^212^Pb (highest *p* = 0.0345; Figure 6a). Six hours after administration, the % ID/g was still significantly higher for ^212^PbCl_2_ in the kidneys, femur, and skull (highest *p* = 0.0189; Figure 6b). After 24 h—that is, more than two physical half-lives of ^212^Pb, a statistically significant difference was detected only in the skull (*p* = 0.0187; Figure 6c). The % ID/g of ^212^Pb in the skeleton appeared to increase with respect to the earlier time points for the MPs. 

In the study comparing ^212^Pb-CaCO_3_ MPs to SMPs, only the 2 h point was evaluated. No statistically significant differences were detected for any of the tissues measured (Figure 6d). However, SMPs resulted in smaller particle aggregates in vivo, which were more widely distributed throughout the abdominal cavity.

### 3.4. Therapeutic Effect of ^212^Pb-CaCO_3_ MPs

**Figure 7:**
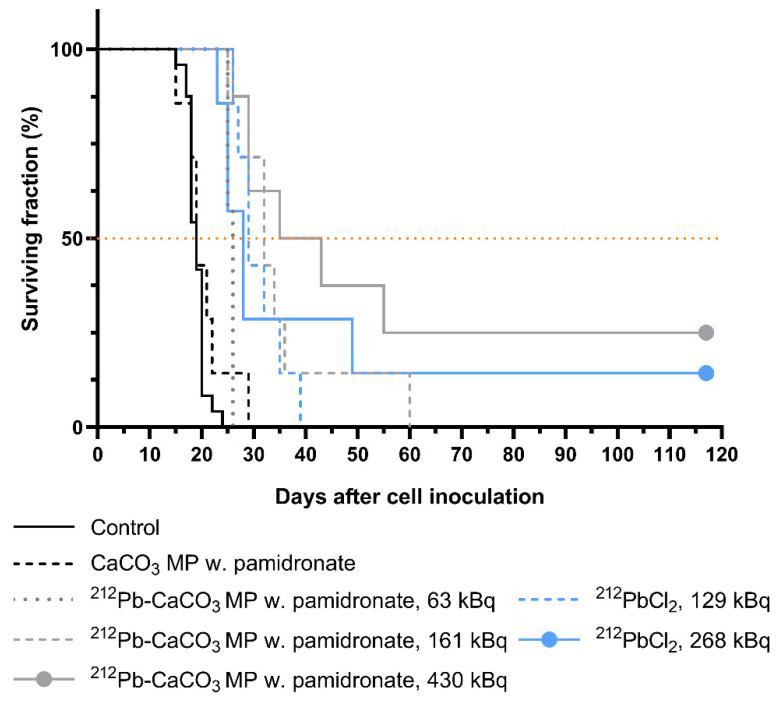
Kaplan–Meier curves of mice bearing ovarian cancer xenografts inoculated intraperitoneally on day 0 and treated with various i.p. treatments on day 1. Two mice were censored from the gray curve, and one mouse was censored from the blue curve due to long-term survival (beyond the study’s conclusion defined as 3× the longest median survival time). Control group (pooled group from three separate experiments): *n* = 24; 430-kBq dose group: *n* = 8; all other experimental groups: *n* = 7.

The therapeutic effect of varying doses of unbound and CaCO_3_ MP–bound ^212^Pb was assessed by comparing the survival curves of xenograft-bearing mice (Figure 7) and adjusting all the obtained *p*-values for multiple comparisons. A significant therapeutic effect was observed for all the tested doses of ^212^Pb-CaCO_3_ MPs and ^212^PbCl_2_ compared to the saline control (highest *p* = 0.0034). Furthermore, a statistically significant survival benefit was achieved with all ^212^Pb treatments compared to non-labeled CaCO_3_ MPs (highest *p* = 0.0366) except for the 63-kBq MP (*p* = 0.1304) and 268-kBq ^212^PbCl_2_ groups (*p* = 0.0825). The therapeutic effect appeared to be dose-dependent for the ^212^Pb-labeled MPs, as the median survival time correlated with the dose. However, a statistically significant difference was observed only between the highest and the lowest dose groups (*p* = 0.0134). A dose–response relationship was not detected for the two ^212^PbCl_2_ doses tested. Statistical significance was not detected for any of the other ^212^Pb treatments, except for the 63-kBq MP group compared to the 129-kBq ^212^PbCl_2_ group (*p* = 0.0249).

The body weight (Supplementary Figure S1) and general condition of the animals in the first two weeks following the treatments indicated no acute treatment-related toxicity in any of the study groups. A slight weight loss (1–2%) was observed in the 430-kBq ^212^Pb-CaCO_3_ MP group during the first days after the treatment, but the body weight returned to baseline within one week.

## 4. Discussion

The application of ^212^Pb in cancer therapy has focused on developing strategies for TAT by coupling ^212^Pb with various tumor-targeting molecules, mostly mAbs. This interest has been accelerated by a combination of the availability of ^212^Pb through commercial ^224^Ra/^212^Pb generators and the simple and efficient labeling of biomolecules through the “custom” bifunctional lead chelator TCMC. In contrast to such active targeting, the use of micro- and nanoparticles as carrier vehicles of ^212^Pb is based on the presumption that it is possible to irradiate tumors without tumor-specific binding, provided that the particles reach the proximity of tumor cells. Particles may overcome several of the limitations of mAbs and peptides, including radiolytic damage to organic molecules, immunogenicity, release of daughter nuclides in vivo, and complex and costly production methods.

Calcium carbonate microparticles, in particularly vaterite particles, are considered suitable as drug delivery vehicles because of their biocompatibility and porous morphology [46]. Therefore, vaterite microparticles could be useful carriers of ^212^Pb in internal alpha therapy provided that they retain the radionuclide in vivo. In this work, the use of pamidronate as a stabilizing additive fixed both the size and vaterite structure of the particles once they were autoclaved, resulting in a uniform suspension of dispersed particles. In contrast, in the absence of pamidronate, the particles recrystallized to calcite immediately after autoclaving, yielding a flaky suspension that settled quickly. As a phosphonate, pamidronate binds calcium and is therefore adsorbed on the particles, as shown by EDX. However, due to the penetration depth of X-rays, it is unknown whether it binds to the surface or in the porous interior of the particles. Nevertheless, unlike the chemically similar EDTMP, pamidronate does not interfere with ^212^Pb adsorption on the particles.

This work demonstrates that various CaCO_3_ particles ranging from vaterite MPs and SMPs to the considerably larger and clustered calcite particles, can be effectively labeled with ^212^Pb in a short and simple process that relies only on sorption of the radionuclide without the need for co-precipitants or chelators. A limitation with the present method is that for clinical application, the labeling should be performed on-site. Thus, we focused on devising a protocol for labeling that can be easily implemented by nuclear medicine personnel at hospital sites. The proposed method involves injecting a ready-to-use ^212^Pb solution directly into a sealed vial of sterile CaCO_3_ microparticles in suspension, whereafter the product is ready for quality control and subsequent use within a few minutes. The labeling yield was almost quantitative, and this fraction of adsorbed ^212^Pb remained stable within two physical half-lives. Although calcite particles were considered unsuitable as ^212^Pb carriers due to their rapid sedimentation rate and nonporous structure, it was of interest to compare the degree of ^212^Pb retained on calcite with vaterite particles. The improved ^212^Pb stability on vaterite MPs and SMPs observed in the in vitro assessment may be attributable to their larger surface area and high porosity, which is advantageous for effective radionuclide sorption, although this was not reflected in the RCP. 

To evaluate the stability and efficacy of ^212^Pb-labeled vaterite MPs and SMPs in vivo, the labeled particles were injected into the peritoneal cavities of mice. The biodistribution study showed that CaCO_3_ MP- and SMP-bound ^212^Pb was more likely to remain intraperitoneally, as less ^212^Pb reached the blood, kidneys, spleen, and skeleton than intraperitoneally administered ^212^PbCl_2_. Although the fact that ^212^Pb accumulated in the kidneys at up to 15% ID/g for CaCO_3_-bound ^212^Pb is significant, the reduction to up to 66% compared to ^212^PbCl_2_ shows that the particles are relatively stable, which is important considering the pharmacokinetic profile of lead and related toxicity concerns [26]. After 24 h, the accumulation in the kidneys decreased to a considerably lower level compared to previously published data on non-specific mAbs and mAb fragments administered intraperitoneally to mice with i.p. tumors [10,12]. 

The pilot study on the therapeutic efficacy of MP-bound and unbound ^212^Pb in mice with i.p. ovarian cancer was conducted using relatively low ^212^Pb doses compared to previous studies on i.p. treatment of mice [8,9,10,11,12,32,33,47], in which multiples of 10 μCi (370 kBq) are typical doses. All the doses tested in this work resulted in a survival benefit compared to control animals. The ^212^Pb-CaCO_3_ MPs did not result in significantly improved survival compared to the i.p. treatment with ^212^PbCl_2_ despite the shorter i.p. retention of the unbound variant. A previous study on ^224^Ra-labeled CaCO_3_ microparticles showed that particle-bound ^224^Ra was significantly more efficacious than ^224^RaCl_2_, even when the latter was given at a 25% higher dose, which was attributed to rapid leakage from the peritoneal cavity and subsequent free radionuclide accumulation in the bones [37]. However, a similar median survival time was achieved with ^212^Pb-CaCO_3_ MPs at only 24–49% of the ^212^PbCl_2_ dose (26 compared to 28 and 29 days). Another important aspect is the reduced non-target exposure (i.e., systemic exposure) when using ^212^Pb-CaCO_3_, which reduces the risk of undesired irradiation of normal tissues. The therapeutic effect of ^212^Pb-CaCO_3_ SMPs was not evaluated in this work, but SMPs are potentially advantageous because of their smaller size, which may provide better distribution of particles within the body cavity, as was observed in necropsies of mice in the biodistribution study. Since neither the MPs nor SMPs have any tumor-targeting moiety, it is important to ensure that the particles are widely distributed inside the body cavity for an optimal coverage of the radioactivity dose. Future studies should therefore aim at evaluating the therapeutic efficacy of SMPs and compare it to that of MPs.

Calcium carbonate particles represent a suitable carrier vehicle for various radionuclides. Successful labeling has been reported for the two alpha-emitting radionuclides ^224^Ra (*t*_1/2_ = 3.6 days) and ^225^Ac (*t*_1/2_ = 9.9 days), and for the positron emitters ^89^Zr (*t*_1/2_ = 78.4 h) and ^68^Ga (*t*_1/2_ = 67.7 min) [48,49,50]. Both ^224^Ra and ^225^Ac are relatively long-lived radionuclides compared to ^212^Pb, which permits a longer production process and shelf-life of the labeled particles before the radiopharmaceutical reaches the end user. On-site labeling is needed for ^212^Pb. The ^224^Ra-CaCO_3_ microparticles (Radspherin^®^) are the predecessors of the ^212^Pb-CaCO_3_ MPs and SMPs developed in this work, with which they share many traits: ^212^Pb is also adsorbed on these particles following the decay of ^224^Ra, and EDTMP is used as a recrystallization inhibitor. The most notable difference is the encapsulation of the particles with an outer layer of CaCO_3_ after they are labeled with ^224^Ra to prevent ^212^Pb-EDTMP complexation. In theory, the layer encapsulation step might be redundant if EDTMP were replaced with pamidronate, as was done in this work. However, such encapsulation could also serve as a means of capturing recoiling daughter nuclides during decay, which is relevant to consider for the alpha emitters that have additional alpha-emitting daughters to prevent redistribution in vivo and its associated adverse effects. In the ^225^Ac micro- and sub-microparticles described by Muslimov et al., CaCO_3_ core–shell particles were synthesized with the radionuclide bound to DOTA-functionalized human serum albumin and incorporated in the particle bulk, followed by layer-by-layer coating of the particles with a protein and polyphenol complex to stabilize them and inhibit recrystallization [48,49]. Like the use of glycerol to reduce the particle size in this work, ethylene glycol was used to obtain submicron particles of 620 nm in diameter. The various CaCO_3_ particles and labeling strategies enable the use of different therapeutic radionuclides and application areas. For instance, a particle size down to the nanometer scale allows intravenous and/or intratumoral administration routes. 

## 5. Conclusions

Calcium carbonate vaterite microparticles represent a promising approach to delivering ^212^Pb to intracavitary tumors. This work demonstrates that ^212^Pb-labeling of vaterite particles in a suspension can be performed in a simple, fast, and efficient way without the need for co-precipitants or chelators. Preclinical results from pilot studies on mice showed that the microparticles retained the ^212^Pb within the peritoneal cavity and that the particle-bound ^212^Pb produced an anti-tumor effect even in low radioactivity doses.

## 6. Patents

A patent application has been submitted for the novel methods and products presented herein.

## Data Availability

The data presented in this study are available from Oncoinvent AS, although they are not publicly available due to availability restrictions under the license of the presented work. However, data are available from the authors upon reasonable request and with the permission of Oncoinvent AS.

## Supplementary Materials

The following are available online at https://www.mdpi.com/article/10.3390/ma14237130/s1. Figure S1: Normalized body weight in the therapeutic efficacy study.
