# Supplementary material for: A Novel Single-Step-Labeled 212Pb-CaCO3 Microparticle for Internal Alpha Therapy: Preparation, Stability, and Preclinical Data from Mice
Source: Materials (Basel). 2021 Nov 23;14(23):7130. doi: 10.3390/ma14237130 (PMC8658347; doi:10.3390/ma14237130)
Supplement: Supplementary file 1 [file materials-14-07130-s001.zip › materials-1463196-supplementary.pdf]

materials

Supplementary Materials to A Novel Single-Step-Labeled $^{212}Pb-CaCO_3$ Microparticle for Internal Alpha Therapy: Preparation, Stability, and Preclinical Data from Mice

Ruth Gong Li[1,2,3,*], Kim Lindland[1], Tina Bjørnlund Bønsdorff[1], Sara Westrøm[1], and Roy Hartvig Larsen[1]

1 Oncoinvent AS, 0484 Oslo, Norway
2 Institute of Clinical Medicine, University of Oslo, 0316 Oslo, Norway
3 Department of Radiation Biology, Institute of Cancer Research, The Norwegian Radium Hospital, Oslo University Hospital, 0379 Oslo, Norway
* Correspondence: li@oncoinvent.com (R.G.L.)

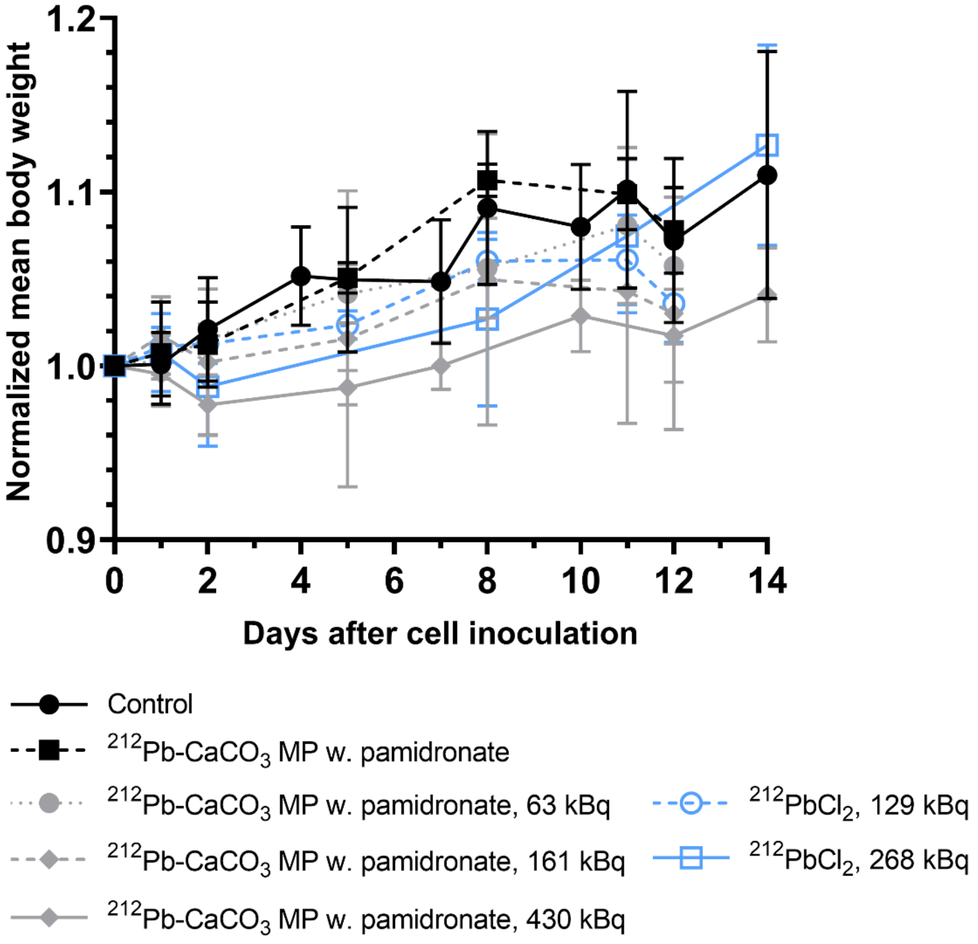

Supplementary Figure S1. Normalized body weight in the therapeutic efficacy study, presented as the mean and standard deviation of each study group with respect to the baseline body weight on day 0. Treatments were administered on day 1.
